# Modelling the energy harvesting from ceramic-based microbial fuel cells by using a fuzzy logic approach

**DOI:** 10.1016/j.apenergy.2019.113321

**Published:** 2019-10-01

**Authors:** Alberto de Ramón-Fernández, M.J. Salar-García, Daniel Ruiz-Fernández, J. Greenman, I. Ieropoulos

**Affiliations:** a Bristol BioEnergy Centre, Bristol Robotic Laboratory, Block T, UWE, Bristol, Coldharbour Lane, Bristol BS16 1QY, UK; b Department of Computer Technology, University of Alicante, Alicante 03690, Spain

**Keywords:** Microbial fuel cells, Ceramic membranes, Fuzzy inference system, Bioenergy, Modelling

## Abstract

Microbial fuel cells (MFCs) is a promising technology that is able to simultaneously produce bioenergy and treat wastewater. Their potential large-scale application is still limited by the need of optimising their power density. The aim of this study is to simulate the absolute power output by ceramic-based MFCs fed with human urine by using a fuzzy inference system in order to maximise the energy harvesting. For this purpose, membrane thickness, anode area and external resistance, were varied by running a 27-parameter combination in triplicate with a total number of 81 assays performed. Performance indices such as R^2^ and variance account for (VAF) were employed in order to compare the accuracy of the fuzzy inference system designed with that obtained by using nonlinear multivariable regression. R^2^ and VAF were calculated as 94.85% and 94.41% for the fuzzy inference system and 79.72% and 65.19% for the nonlinear multivariable regression model, respectively. As a result, these indices revealed that the prediction of the absolute power output by ceramic-based MFCs of the fuzzy-based systems is more reliable than the nonlinear multivariable regression approach. The analysis of the response surface obtained by the fuzzy inference system determines that the maximum absolute power output by the air-breathing set-up studied is 450 μW when the anode area ranged from 160 to 200 cm^2^, the external loading is approximately 900 Ω and a membrane thickness of 1.6 mm, taking into account that the results also confirm that the latter parameter does not show a significant effect on the power output in the range of values studied.

## 1 Introduction

Microbial fuel cells (MFCs) is an environment-friendly technology, which benefits from bacterial metabolism to produce clean energy. Fossil fuel combustion is still practised to meet the global energy demand; however, their depletion has encouraged the search for alternative energy sources. In order to address the environmental challenges caused by global warming, as well as fossil fuel depletion, innovative and powerful technologies such as MFCs have emerged in recent years [1], [2], [3], [4].

An MFC consists of an anodic and cathodic chamber physically separated by a membrane. In the anode, bacteria oxidise the organic matter contained in a specific substrate, releasing protons and electrons. Protons diffuse from the anode to the cathode via the membrane and along with incoming electrons, flowing from the anode through an external circuit, they recombine to produce water. The anodic oxidation reaction is balanced by a reduction reaction at the cathode, where oxygen usually acts as an electron acceptor. In order to accelerate the oxygen reduction reaction, catalysts such as platinum are commonly employed. One of the main benefits of this technology is to use complex substrates, such as domestic or industrial wastewater as fuel, allowing the system to treat wastewater and generate electricity simultaneously [5], [6], [7].

Despite the potential of MFCs, they still have some limitations, which hinder their large-scale commercialisation, one being energy density. This very much depends on the electrode material, the nature of the separator, the set-up or the operating conditions, among others [8], [9]. Since MFCs are complex systems, their optimisation becomes a key challenge to deal with. In recent years, significant effort has been made in terms of architecture, design and stack configurations in order to both maximise the power output and reduce the overall cost of the system [10], [11]. However, experimental work is often too costly, time-consuming, and rarely represents real world conditions, which collectively limit the progress of the technology. For all these reasons, the use of modelling tools for both optimising and predicting the performance of MFCs has gained attention in the last few years [12], [13], [14], [15]. These techniques address multiple scenarios simultaneously, being able to cover extreme conditions that are difficult to assay. Mathematical models are usually based on differential and algebraic equations, which focus on multiple phenomena that take place in MFCs. Since they are complex systems, these models need an in-depth understanding of the internal MFC processes. Mathematical models can be grouped into conventional and non-conventional. The most implemented are the conventional models, which usually describe phenomena that take place in the anodic or cathodic chamber such as kinetic reactions, biofilm growth, mass transfer through the membrane or electrochemical principles [15]. Recently the use of a numerical approach based on an optimised formulation of Boltzmann’s kinetic equation has been reported. In this case, the authors simulated the power and polarisation curves obtained by MFCs fed in batch mode, with the solid fraction of municipal waste by using as input variables the pH, bacterial activity and current density [16]. Previously, the same authors applied similar methodology also to predict the behaviour of MFCs fed with vegetable waste in terms of power and polarisation curves. They tracked the evolution over time of three different species simultaneously, and using their code, they were able to achieve ≈1.5 million lattice sited updates per second [17].

On the other hand, alternative analysis systems based on non-specific processes have also emerged recently, with artificial intelligence (AI) being one of the most promising. AI allows us to develop intelligent software in order to solve specific problems in a broad domain such as health, business, biotechnology, etc. This modelling tool is able to detect hidden interactions between input and output variables, which brings enormous benefits in data-saturated domains, with improved accuracy [18]. AI-based models, which include Neural Networks (NN), Fuzzy Logic (FL) or Neural-Fuzzy methods (NF) are useful for designing a pattern of behaviour in nonlinear systems [19]. In this kind of modelling tools, the data are used for both creating the model and confirming its accuracy. Their main advantage is that they can correlate input and output variables in a given system without in-depth knowledge of their behaviour. Fuzzy logic is a mathematical approach introduced by Zadeh in 1965 [20], in which the truth value of the variables may be any real number between 0 and 1 and can range between completely false and completely true. This technique provides an inference system able to replicate the human reasoning procedures in knowledge-based systems. Conventional Boolean logic grouped the information as totally true or totally false, however in real life not all variables can be expressed with this level of certainty. Fuzzy logic allows us to work with vague information as well as mathematically depict the uncertainty of something being either completely true or completely false [21], [22].

In the last few years, the number of research reports focussing on demonstrating the implementation of MFCs into practical applications, has significantly increased [23], [24], [25], [26]. As a result, it has been reported that MFCs can provide sufficient power for a mobile robot to perform photo-taxis [27]. MFCs have also successfully powered a meteorological buoy during a long-term deployment [28]. With regard to ceramic-based MFCs fed with human urine, recent research articles also report the feasibility of this technology to recharge devices such as mobile phones. In particular, the energy harvesting from urine-fed MFCs is able to charge up to 3.7 V in 24 h the battery of a mobile phone [29]. This study confirms the feasibility of using urine as renewable fuel for generating useful bioenergy through MFCs. More recently, further improvements were reported by Walter *et al*. regarding the use of ceramic-based MFCs fed with 600 mL of urine, which concluded that a smartphone can be functioning during 3 h (including calls) after being charged over 6 h [30]. These results confirm that the technology is feasible for out-of-the-lab applications. However, in order to get the most out of these devices, it is crucial to optimise their performance.

In this context, the novelty of this work is the modelling of ceramic-based MFCs fed with human urine in order to maximise the energy harvesting, which will facilitate the practical implementation of this biotechnology. For this purpose, a fuzzy logic approach is used to model the effect of different design and operating parameters, such as anode area, ceramic thickness and external resistance on the power output by an air-breathing system. In particular, in this work a fuzzy inference system is developed from the fuzzification of the three input variables, the knowledge base formation and the defuzzification of the output values obtained by the fuzzy inference calculations. Eventually, the predicted values and the experimental results were compared, but it was also possible to predict new data not derived experimentally. The efficiency of the model designed is evaluated in terms of the predictive capability of the experimental results. To the best of the authors’ knowledge, the existing literature which focuses on modelling ceramic-MFCs by AI is limited or non-existent.

## 2 Materials and methods

### 2.1 MFC assembly

**Fig. 1:**
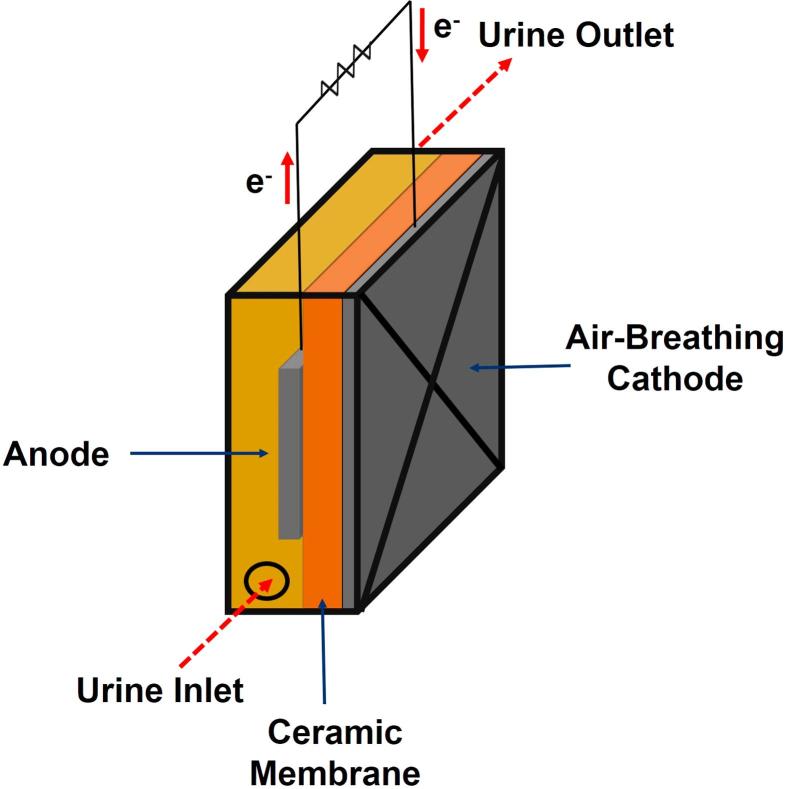
Ceramic-based MFC set-up assessed.

The MFC set-up assessed was a cubical air-breathing design (see Fig. 1). The anode consists of carbon veil (30 g m^−2^, PRF composites, Dorset. UK) coated with activated carbon (AC. GBaldwin&Co. UK), whereas the cathode is made of a blend of AC-PTFE (80-20) pressed over a stainless steel mesh. Flat membranes were handmade by kilning square pieces of wet terracotta clay for 3 min at 1070 °C and a ramp time of 7 h. The final size of the square membranes was 3 cm × 3 cm. MFCs were initially inoculated with a mixture of sludge and fresh urine (1:1 v/v) in batch mode. After 4 days in which the fuel was completely replenished every day, the MFCs were continuously fed with fresh urine at a flow rate of 0.1 mL min^−1^.

In order to optimise the performance of the MFC set-up, the effect of three different design and operating parameters on the power performance was experimentally assessed. These results were used to obtain a fuzzy logic model, which will allow us to predict the behaviour of the system. Three different levels for anode area (22.25, 102.25 and 182.25 cm^2^), membrane thickness (1, 1.6 and 2.2 mm) and external resistance (20, 710 and 1400 Ω) were assessed. The voltage was continuously monitored by an Agilent data logger (LXI 34972A data acquisition/Switch unit) during 360 h.

The selection of the parameters to study was made on the basis of previous experimental tests run with alternative ceramic-MFCs set-up [31], [32]. These results reported that anode area, membrane thickness as well as the external loading might have significant effect on the performance of ceramic MFCs. For these reasons, these same parameters were selected as a starting point for the optimisation of the ceramic-MFCs set-up designed in this work, contemplating the possibility of applying the same methodology to other parameters whose effect on the power output of other design of MFCs has also reported in literature [33] and will help us to better understand the behaviour of the system.

### 2.2 Fuzzy inference system

**Fig. 2:**
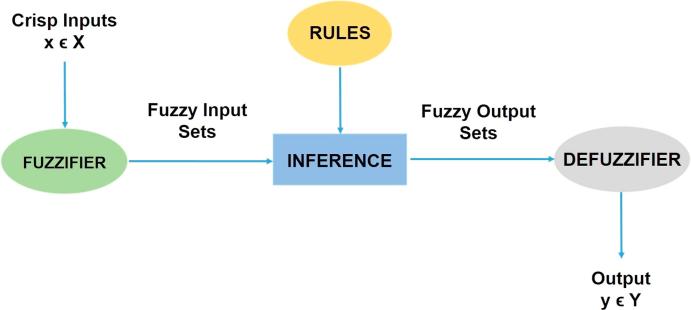
General structure of a fuzzy logic system.

Fuzzy inference systems (FIS) are systems with approximate reasoning whereby possible conclusions are deduced from incomplete information [34]. Currently, there are two types of FIS widely used, named Sugeno and Mamdani [35], [36]. The general architecture of Mamdani FIS can be divided into three stages: (i) fuzzification of the input variables, (ii) inference or rule processing and (iii) defuzzification of the variables and release of the output. Fig. 2 shows this process.

#### 2.2.1 Fuzzification

Fuzzification consists of coding discrete values (crisp values) to values of a fuzzy set by assigning a certain degree of membership. Therefore, a fuzzy set “A” is a class of objects “x” of the same space of points “X” with a continuum of membership degrees to that set [20]. This process allows us to evaluate a proposition as partially true or false. This degree of membership is depicted by a number in the interval [0,1] and is determined by the membership function $\mu_{A}$ that defines the fuzzy set:

(1) $$\mu_{A}(x)\to [0,1],$$

where $\mu_{A}(x)$ = 1 if *x* belongs wholly to the set *A*, $\mu_{A}(x)$ = 0 if *x* does not belong to the set *A* and $\mu_{A}(x)$<1 if *x* belongs partially to the set *A*. Depending on the problem to be treated, the membership function is particularised for each variable. The most commonly used membership functions are: triangular, trapezoidal, Gaussian and sigmoidal. All of them must be continuous and take values between 0 and 1. To create our fuzzy system, Gaussian and sigmoidal functions have been chosen to represent the input and output variables, since they adjust better to the real behaviour of our variables than the rest of the functions. The sigmoidal function is defined by its lower limit a, upper limit b and the m value or inflection point, such that *a*<*m*<*b*. The slope of the curve increases as the (a - b) distance also increases (see Fig. S1a in supplementary information).

On the other hand, Gaussian function performs a normal distribution of a continuous variable. It is defined by its average value *c* and the variance $\sigma^{2}$ (see Fig. S1b in supplementary information).

#### 2.2.2 Inference system

**Fig. 3:**
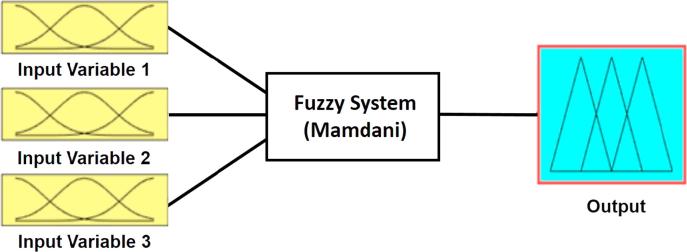
General structure of a three-input and one-output Mamdani system.

The inference unit is responsible for generating an output value for each input value using the fuzzy sets theory [20]. In this work, a Mamdani FIS has been used since offers a greater expressive power and interpretability than Sugeno system [37]. Fig. 3 shows the general structure of a three-input and one-output Mamdani system.

The inference process applies fuzzy rules to the inputs after the fuzzification. A fuzzy relationship represents the degree of presence or absence of association between two or more fuzzy set processes [38], [39]. Fuzzy rules are responsible for modelling the problem that needs to be solved and they are expressed as a relationship between antecedent (IF) and consequent (THEN):

(2) IF<fuzzyantecedent>THEN<fuzzyconsequent>,

where <*fuzzy antecedent*> and <*fuzzy consequent*> can be an atomic sentence (single) or multiple. Each rule is evaluated by the system independently, obtaining a value of the consequent according to the truth value of the antecedent.

For a better understanding of the rules evaluation process, an example based on the inference of two fuzzy rules is described (see Example S1 in supplementary information).

#### 2.2.3 Defuzzification

The result must be expressed in a crisp value, being the centroid method one of the most used for this purpose [40]. This method calculates the discrete value whose vertical divides the fuzzy output set into two equal areas (see Fig. S2d in supplementary information).

## 3 Results and discussion

The aim of this study is to provide a fuzzy logic model to predict the power performance of MFCs. To this end, a fuzzy logic system based on three-input variables and one-output variable is implemented by using MATLAB fuzzy logic toolbox in Windows 10. According to the Mamdani-based scheme shown in Fig. 3, input variable 1, 2 and 3 are the membrane thickness (mm), the external resistance (Ω) and the anode area (cm^2^), respectively.

**Fig. 4:**
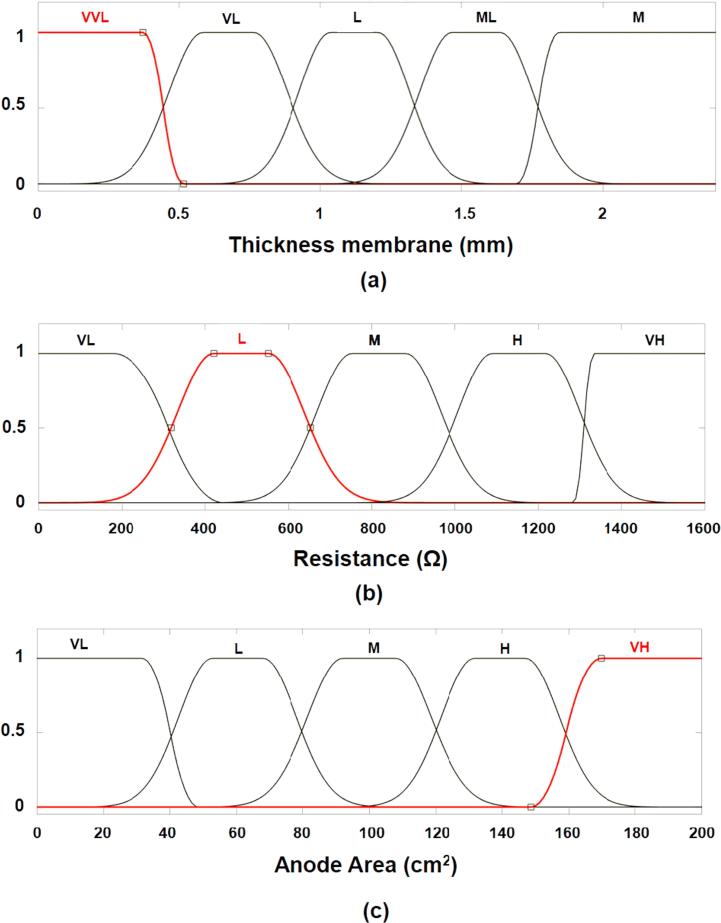
Fuzzy membership functions for input variables: (a) Thickness fuzzy set plot; (b) Resistance fuzzy set plot and (c) Anode area fuzzy set plot.

**Fig. 5:**
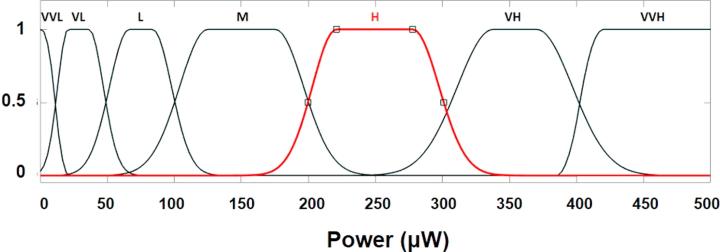
Fuzzy membership functions for output variable: Power fuzzy set graphic.

The fuzzy set of the input and output variables has been defined by their membership functions (see Fig. 4, Fig. 5). These membership functions represent the fuzzification as linguistic variables of the numerical parameters of input and output variables. Input variables 2 and 3 have been defined by five linguistic variables: very low (VL), low (L), medium (M), high (H) and very high (VH), whereas input variable 1 was defined as very very low (VVL), very low, low, medium low (ML) and medium. On the other hand, in order to obtain more accuracy, the output variable has been defined by seven linguistic variables: very very low, very low, medium, high, very high and very very high (VVH). The sigmoidal function and a variant of the Gaussian function were used to define the fuzzy set. As already mentioned, Gaussian function depends on two parameters, $\sigma^{2}$ and c, as given by $f\left(x,\sigma_{c}^{2},c\right)=e^{\frac{-{\left(x-c\right)}^{2}}{2\sigma^{2}}}$. The first function, specified by $\sigma_{1}^{2}$ and ${\text{c}}_{1}$, determines the shape of the left-most curve. The second function specified by $\sigma_{2}^{2}$ and c_2_ determines the shape of the right-most curve. Whenever c_1_< c_2_, the Gaussian function reaches a maximum value of one. Otherwise, the maximum value is less than one. Table S1 (in supplementary information) displays the parameters for the construction of the functions. It should be noted that these parameters have been empirically selected.

For the prediction of MFCs power performance, three variables have been analysed. These variables were classified according to five linguistic variables. This means a total of 125 possible combinations or case studies. For each case of study, a power value encoded according to seven linguistic variables is obtained. The fuzzy system interprets independently each case of study as a rule of inference. A fuzzy system will be better defined as the number of fuzzy rules increases. However, it is not always possible to simulate all the possible study cases, either because the experimental data are not available or because the number of possible combinations is too large. That is why the system (thanks to the rules introduced and the fuzzy sets defined for each variable), will be responsible for inferring the rest of the cases. Thus, the number of inference rules introduced as well as the precision in the definition of the membership functions are key factors for the performance of the fuzzy system. In our case, a total number of 60 inference rules have been defined. Some of these rules are defined in Table S2 (in supplementary information).

In order to assess the computational cost, multiple simulations of the proposed fuzzy logic system have been carried out. The fuzzy algorithm has been run 500 times and the computational cost is measured by its computational time (CT), obtained from MATLAB tic and toc functions. These functions allow to estimate how long a portion of code takes to run. The simulations were performed on a personal computer with a single processor (Intel Core i5-4210U CPU 2.40 GHz processor, 6.00 GB RAM), and software package MATLAB R2017b in Windows 10. When performed on a single-processor machine, the computational cost will depend on the complexity of the fuzzy algorithm itself and the speed of movement of the data between different components of the memory. The obtained average, maximum and minimum CT were 0.01100712, 0.298841, 0.00332544 s, respectively. These figures show a very short and reasonable computational time for the fuzzy algorithm in a single processor computer, thus being feasible for a real-time application. Compared with the mathematical models reported in literature such as those 1-D,2-D and 3-D developed by Picioreanu et al. [12] in which the computational cost is 6 min, 30 min and 14 h respectively, the fuzzy approach employed in this work has a meaningful advantage.

**Fig. 6:**
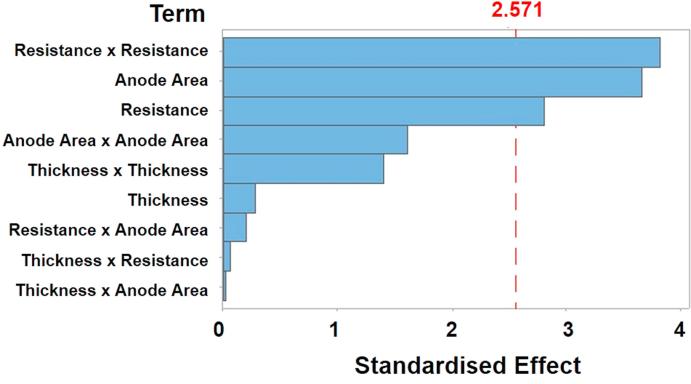
Pareto chart of the standardised effects.

In addition to the fuzzy inference system constructed in this work, a nonlinear multivariable regression analysis was also carried out to compare the results obtained by the fuzzy system. In this analysis, both p-value and f-value were employed as statistical parameters for analysing the significance of the variables with a 95% confidence (p < 0.05). Fig. 6 shows Pareto chart which depicts the standardised effects with p = 0.05. The bar length belongs to the absolute standardised value. Only the bars related to both factors, external resistance and anode area, as well as the quadratic interaction of the resistance overcome the reference line (2.571), being the only effects statistically significant. The significant contribution of the resistance quadratic effect reports the presence of a curvature over the response surface associated with the model.

The analysis of the variance in power allows us to obtain a simplified second order model equation:

(3) $$P=-143.7+0.693\times \mathrm{Resistance}+1.595\times \mathrm{Area}+0.000388\times {\mathrm{Resistance}}^{2}$$

In order to compare both methods, some statistical indices such as R-squared (R^2^) and variance account for (VAF) were calculated. R^2^ evaluates the goodness-of-fit of the predicted values versus the observed values. The value of R^2^ ranges between 0 and 100% and the higher the value of R^2^, the tighter fit of the measured values to the model. VAF is commonly used to verify the accuracy of a model, by comparing the real output with the estimated output by the model. The VAF of two data sets that are the same is 100%, whereas if they are different, VAF will be lower. Table 1 contains the value of each statistical parameter analysed for both fuzzy inference system and nonlinear multiple regression. In the case of fuzzy inference system, both R^2^ and VAF are very close to 100% (94.85% and 94.41%, respectively). As mentioned above, it means that there is a tight fit between the observed and the predicted values. However, by using nonlinear multiple regression approach, the value of both parameters decreases up to 79.72% and 65.19% respectively, which indicate a lack-of-fit between the predicted values and the observed values. These results show that the prediction of the ceramic-based MFC power performance is substantially better using the fuzzy logic-based system rather than the nonlinear multiple regression.

**Table 1:** Statistical performance indices.

PerformanceIndex	Fuzzy InferenceSystem	Nonlinear multiple Regression
R2	94.85	79.72
VAF	94.41	65.19

**Fig. 7:**
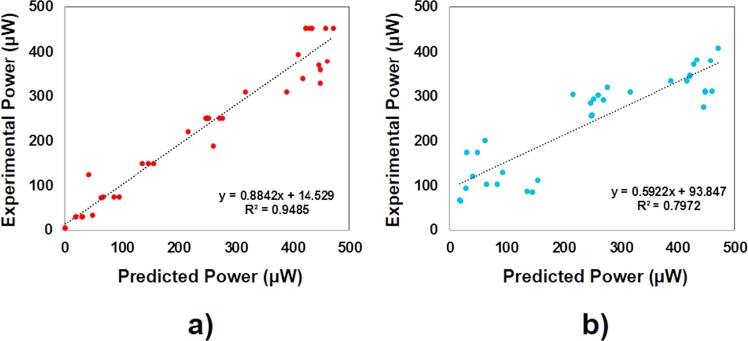
Cross-correlation between the measured absolute power output and the absolute power output predicted by: (a) Fuzzy inference-based system and (b) Nonlinear multivariable regression.

Fig. 7 shows the cross-correlation between the observed absolute power output and the predicted value by using the fuzzy inference-based system (see Fig. 7 and the nonlinear multivariable regression (see Fig. 7b). As can be seen, the fit of the predicted values of the absolute power output to the experimental values is better by using the fuzzy logic-based system compared to the nonlinear multiple regression. In the case of the nonlinear multiple regression system, the lack-of-fit is mainly visible for power output levels lower than 200 μW.

**Fig. 8:**
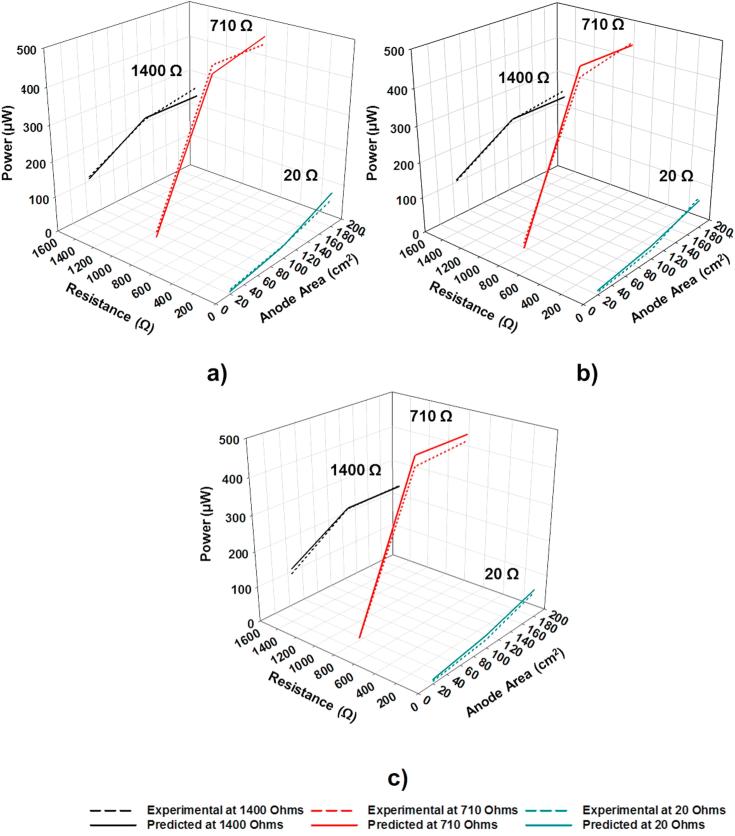
Comparison of the experimental data (solid line) with the power output predicted by using the designed fuzzy inference system (dotted line): (a) Membrane thickness of 1 mm; (b) Membrane thickness of 1.6 mm and (c) membrane thickness of 2 mm.

Fig. 8 shows the comparison between the experimental data and the predicted power output by using the fuzzy inference system designed. As can be observed, the fuzzy-based system allows a more accurate prediction of the absolute power output by ceramic-based MFCs.

**Fig. 9:**
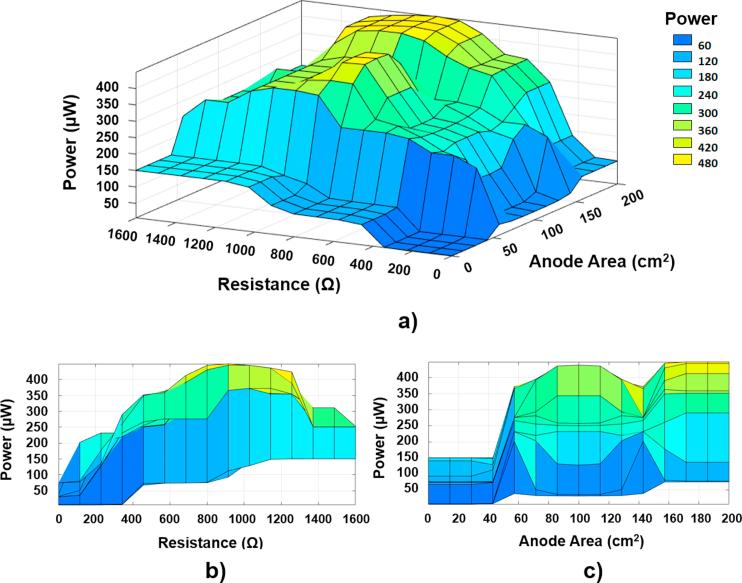
(a) Response surface for the absolute power output by ceramic-MFCs based on the fuzzy logic approach; (b) Power versus Resistance and (c) Power versus Anode Area for a membrane thickness of 1.6 mm.

Furthermore, the fuzzy system designed was also employed for predicting data that were not experimentally assessed. Fig. 9 shows the response surface for the absolute power predicted by the fuzzy-based system. As can be seen, the values that maximise the absolute power output are around an external loading of 900 Ω and anode area of 170 cm^2^. The statistical analysis of the experimental results reports that the influence of the membrane thickness, in the range studied, on the power performance is not significant in comparison with the influence of both the anode area and the external resistance (see Fig. 6). Based on these results, the cross-correlation between the two variables, having the most influence on the model, was investigated in order to analyse the power output behaviour (see Fig. 9a, b and c).

**Fig. 10:**
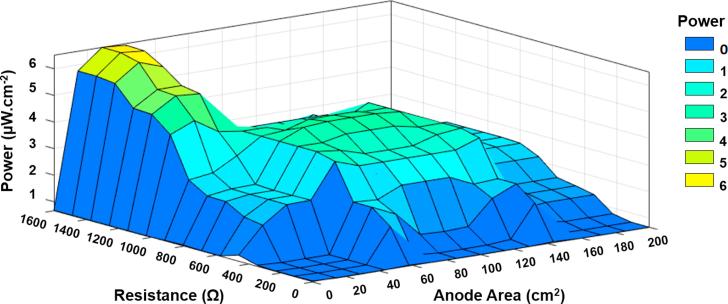
Response surface for the power output normalised to anode area by ceramic-MFCs based on the fuzzy logic approach.

However, it also worth mentioning that from a scaling point of view, the efficiency of the anode is an important parameter to consider. In this case, both observed and predicted values show that the efficiency of the anode increases as its area decreases, being maximum at normalised anode areas smaller than 40 cm^2^ (see Fig. 10).

This work shows the first attempt to model the performance of ceramic-based MFC fed with human urine by using a fuzzy inference system. Unlike previous research reported in literature, which use simple substrates such as glucose or acetate as fuel, in this case real waste is used to both derive the tests and validate the model. This fact renders our system realistic even though the modelling process is much more difficult. The results obtained demonstrate that the fuzzy logic approach employed in this work is a useful tool to maximise the energy harvesting from these devices. The model proposed helps to identify the optimum anode surface area and load value with an accuracy of 94.85%, and therefore save valuable design and set-up time for any practical application.

## 4 Conclusions

The absolute power output by ceramic-MFCs fed with human urine was estimated by using a fuzzy inference system, being 450 μW the maximum value reached by the set-up studied when the anode area ranged from 160 to 200 cm^2^, the external loading is approximately 900 Ω and the membrane thickness is 1.6 mm. The results obtained were also compared with those reported by a nonlinear regression analysis. By using the data collected from 81 runs experimentally assessed, the effect of the anode area, membrane thickness and external resistance on the absolute power output was also analysed. R^2^ and VAF were used as statistical indices to compare the fit of the estimated absolute power output to the observed value. Both parameters show that fuzzy inference system is more reliable to estimate the absolute power output by ceramic-based MFCs than nonlinear multivariable regression. In this case, the fuzzy logic-based model allows us to predict the power performance of this MFC set-up with an accuracy of 94.85%. With regard to inference of parameters not directly explored, the fuzzy inference system allows for a better characterisation of the MFC prototype and model, and consequently a more accurate scaling when such prototypes are designed for practical applications in the real world. The results show that fuzzy inference system is a useful and reliable tool for predicting and modelling the energy harvesting from ceramic-based MFCs, which will facilitate the implementation process of the technology into real application.
