# Supplementary material for: Modelling the energy harvesting from ceramic-based microbial fuel cells by using a fuzzy logic approach
Source: Appl Energy. 2019 Oct 1;251:113321. doi: 10.1016/j.apenergy.2019.113321 (PMC6880661; doi:10.1016/j.apenergy.2019.113321)
Supplement: Appendix A [file mmc1.pdf]

Supplementary Information

Figure S1

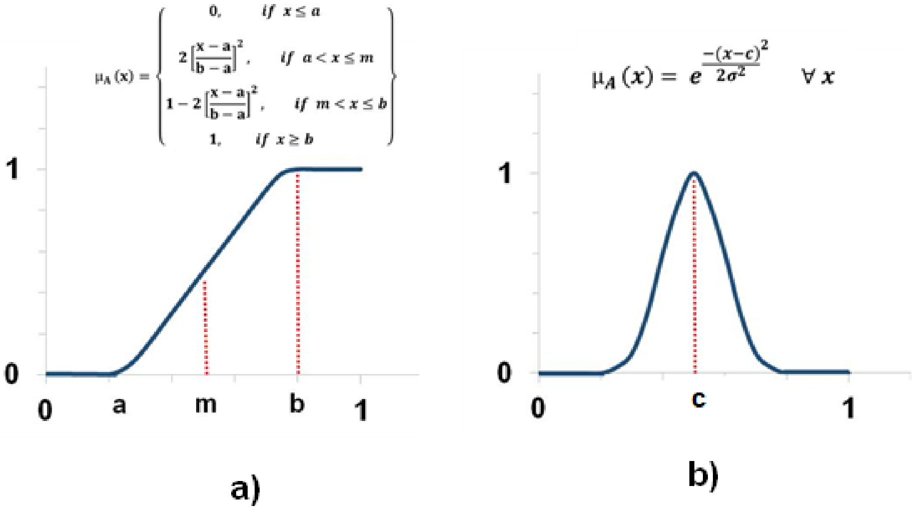

Figure S1: (a) Sigmoidal membership function and (b) Gaussian membership function.

Example S1

A fuzzy system is built by two input variables (x,y) and an output variable (z), whose fuzzy sets (A, B and C) are defined by their corresponding membership functions: μ_A(x), μ_B (y), μ_C(z), respectively (see Figure S2a). Then, each rule is evaluated independently (see Figure S2b):

- *Rule 1: If x1 is A3 OR y1 is B1 THEN z is C1*

  As can be observed, for x=x1 there is no degree of membership to the fuzzy subset A3 ($\mu_{(x=A3)}$=0). However, for y=y1, the degree of membership to the function B1 is 0.1 ($\mu_{(y=B1)}$=0.1). To evaluate the disjunction (OR operator), the standard T-Conorm (maximum) is commonly used, that is, $\mu_A(x) \cup \mu_B(y)$=max[μ_A(x),μ_B(y)]= 0.1. This value indicates the truth value in the response ($\mu_{(z=C1)}$=0.1).

- *Rule 2: If x1 is A2 AND y1 is B2 THEN z is C2*

  In this case, for x=x1 the degree of membership to the function that defines the fuzzy subset A2 is

0.2 ($\mu_{(x=A2)}$=0.2). In the same way, for y=y1 the degree of membership to the function B2 is 0.7 ($\mu_{(y=B2)}$=0.7).In this case, to evaluate the conjunction (AND operator) the standard T-Norm (minimum) is commonly employed, that is, $\mu_A(x) \cap \mu_B(y)$=min[μ_A(x),μ_B(y)]= 0.2. This value indicates the truth value in the response ($\mu_{(z=C2)}$=0.2).

Then, the result of the evaluation of the antecedent is implemented to the consequent, applying a cut according to the truth value of the antecedent. Finally, all the membership functions of all the antecedents combined to obtain a single fuzzy set (see Figure S2c and S2d).

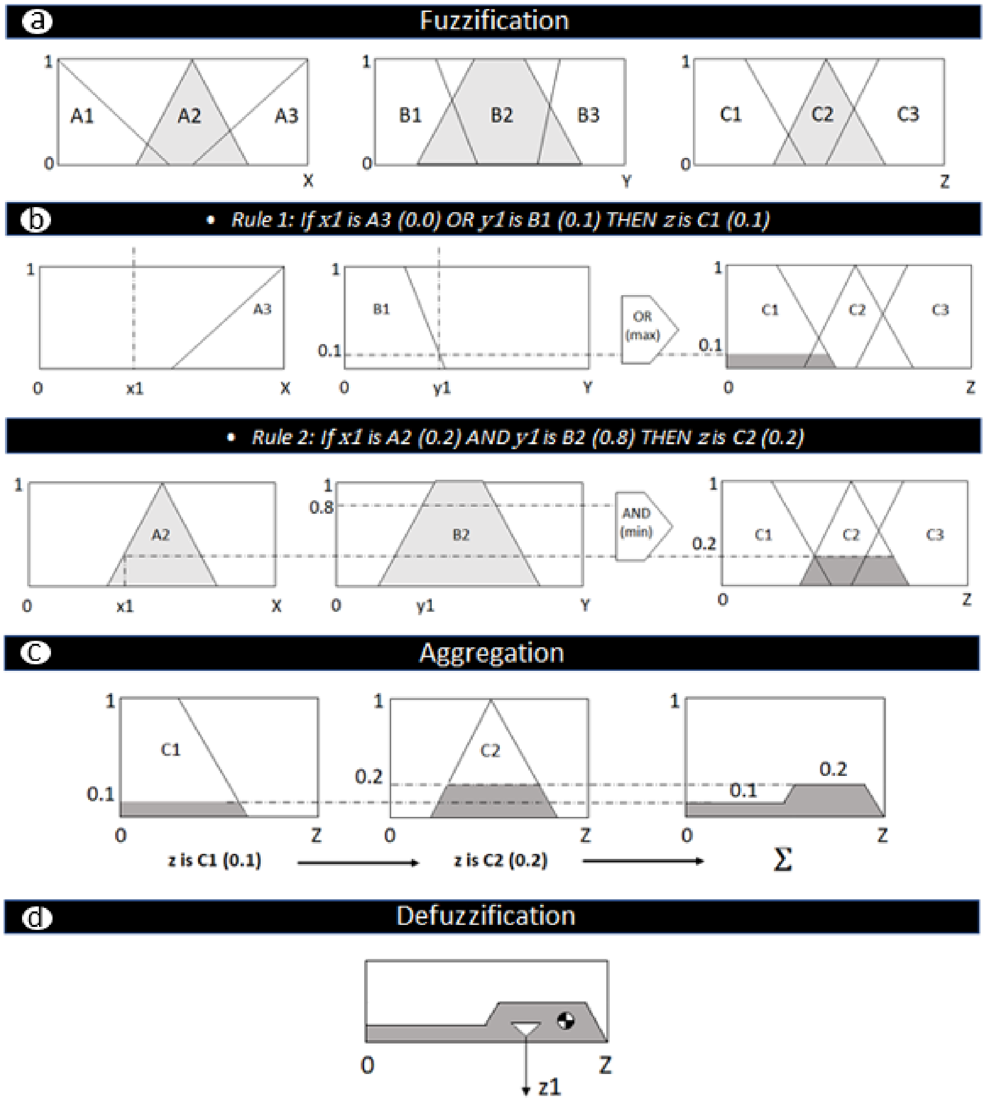

Figure S2: (a) General structure of a Mamdani inference system: (a) Fuzzification; (b) Rules; (c) Aggregation and (d) Defuzzification.

**Thickness, T (Input 1))**	**Function parameters**
Very Very Low (VVL)	μ(x) = sigmf(x,a,c) $\Rightarrow$ μ(x) = sigmf(x, 0.3732, 0.516)}
Very low (VL)	μ(x) = Gauss2mf (x,1,c1, 2,c2) $\Rightarrow$ μ(x) = Gauss2mf (x, 0.1192, 0.5843, 0.1192, 0.7637)}
Low (L)	μ(x) = Gauss2mf (x,1,c1, 2,c2) $\Rightarrow$ μ(x) = Gauss2mf (x, 0.1113, 1.036, 0.1113, 1.204)}
Medium Low (ML)	μ(x) = Gauss2mf (x,1,c1, 2,c2) $\Rightarrow$ μ(x) = Gauss2mf (x, 0.1143, 1.465, 0.1143, 1.637)}
Medium (M)	μ(x) = sigmf(x,a,c) $\Rightarrow$ μ(x) = sigmf(x, 1.695, 1.845)}
**Resistance, R (Input 2)**	**Function parameters**
Very Low (VL)	μ(x) = sigmf(x,a,c) $\Rightarrow$ μ(x) = sigmf(x, 180.3, 444.6)}
Low (L)	μ(x) = Gauss2mf (x,1,c1, 2,c2) $\Rightarrow$ μ(x) = Gauss2mf (x, 86.58, 421.2, 86.58, 551.4)}
Medium (M)	μ(x) = Gauss2mf (x,1,c1, 2,c2) $\Rightarrow$ μ(x) = Gauss2mf (x, 84.69, 753.2, 84.69, 880.6)}
High (H)	μ(x) = Gauss2mf (x,1,c1, 2,c2) $\Rightarrow$ μ(x) = Gauss2mf (x, 83.16, 1090, 83.16, 1216)}
Very High (VH)	μ(x) = sigmf(x,a,c) $\Rightarrow$ μ(x) = sigmf(x, 1283, 1333)}
**Anode area, A (Input 3)**	**Function parameters**
Very Low (VL)	μ(x) = sigmf(x,a,c) $\Rightarrow$ μ(x) = sigmf(x, 31.7, 48.65)}
Low (L)	μ(x) = Gauss2mf (x,1,c1, 2,c2) $\Rightarrow$ μ(x) = Gauss2mf (x, 10.07, 52.83, 10.07, 67.99)}
Medium (M)	μ(x) = Gauss2mf (x,1,c1, 2,c2) $\Rightarrow$ μ(x) = Gauss2mf (x, 10.56, 92.05, 10.56, 107.9)}
High (H)	μ(x) = Gauss2mf (x,1,c1, 2,c2) $\Rightarrow$ μ(x) = Gauss2mf x, 10.13, 131.7, 10.13, 146.9)}
Very High (VH)	μ(x) = sigmf(x,a,c) $\Rightarrow$ μ(x) = sigmf(x, 148.6, 169.7)}
**Power, P (Output)**	**Function parameters**
Very Very Low (VVL)	μ(x) = sigmf(x,a,c) $\Rightarrow$ μ(x) = sigmf(x, 2, 19.9)}
Very low (VL)	μ(x) = Gauss2mf (x,1,c1, 2,c2) $\Rightarrow$ μ(x) = Gauss2mf (x, 7.7, 20.71, 10.7, 36.4)}
Low (L)	μ(x) = Gauss2mf (x,1,c1, 2,c2) $\Rightarrow$ μ(x) = Gauss2mf (x, 14.7, 66.33, 14.2 83.8)}
Medium (M)	μ(x) = Gauss2mf (x,1,c1, 2,c2) $\Rightarrow$ μ(x) = Gauss2mf (x, 21.1, 125, 21.1, 175.3)}
High (H)	μ(x) = Gauss2mf (x,1,c1, 2,c2) $\Rightarrow$ μ(x) = Gauss2mf (x, 18.1, 221, 19.7, 277.7)}
Very High (VH)	μ(x) =Gauss2mf (x,1,c1, 2,c2) $\Rightarrow$ μ(x) = Gauss2mf (x, 26.7, 337.7, 26.7, 370.2)}
Very Very High (VVH)	μ(x) = sigmf(x,a,c) $\Rightarrow$ μ(x) = sigmf(x, 0.359, 404)}

Table S1: Parameters for the construction of the membership functions.

**Rule Number**		**T (mm)**		**R (Ω)**		**A (cm^2)**		**P (μW)**
1	if	L	and	VL	and	VL	then	VVL
2	if	ML	and	VL	and	VL	then	VVL
3	if	M	and	VL	and	VL	then	VVL
4	if	L	and	VL	and	M	then	VL
.	if	.	and	.	and	.	then	.
.	if	.	and	.	and	.	then	.
.	if	.	and	.	and	.	then	.
28	if	L	and	VH	and	VL	then	M
29	if	ML	and	VH	and	VL	then	M
30	if	M	and	VH	and	VL	then	M
31	if	ML	and	L	and	VH	then	M
.	if	.	and	.	and	.	then	.
.	if	.	and	.	and	.	then	.
.	if	.	and	.	and	.	then	.
57	if	L	and	M	and	M	then	VVH
58	if	ML	and	M	and	M	then	VVH
59	if	M	and	M	and	M	then	VVH
60	if	L	and	M	and	VH	then	VVH

Table S2: Fuzzy rules for absolute power output.
